# Splenic T1-mapping: a novel quantitative method for assessing adenosine stress adequacy for cardiovascular magnetic resonance

**DOI:** 10.1186/s12968-016-0318-2

**Published:** 2017-01-13

**Authors:** Alexander Liu, Rohan S. Wijesurendra, Rina Ariga, Masliza Mahmod, Eylem Levelt, Andreas Greiser, Mario Petrou, George Krasopoulos, John C. Forfar, Rajesh K. Kharbanda, Keith M. Channon, Stefan Neubauer, Stefan K. Piechnik, Vanessa M. Ferreira

**Affiliations:** 1 Oxford Centre for Clinical Magnetic Resonance Research (OCMR), Division of Cardiovascular Medicine, Radcliffe Department of Medicine, University of Oxford, Oxford, UK; 2 Siemens Healthcare GmbH, Erlangen, Germany; 3 Department of Cardiothoracic Surgery, John Radcliffe Hospital, Oxford, UK; 4 Oxford Heart Centre, John Radcliffe Hospital, Oxford, UK; 5 Division of Cardiovascular Medicine, Radcliffe Department of Medicine, University of Oxford, Oxford, UK

**Keywords:** Cardiovascular magnetic resonance, Adenosine stress, Splenic T1, Switch-off, ShMOLLI

## Abstract

**Background:**

Perfusion cardiovascular magnetic resonance (CMR) performed with inadequate adenosine stress leads to false-negative results and suboptimal clinical management. The recently proposed marker of adequate stress, the “splenic switch-off” sign, detects splenic blood flow attenuation during stress perfusion (spleen appears dark), but can only be assessed after gadolinium first-pass, when it is too late to optimize the stress response. Reduction in splenic blood volume during adenosine stress is expected to shorten native splenic T1, which may predict splenic switch-off without the need for gadolinium.

**Methods:**

Two-hundred and twelve subjects underwent adenosine stress CMR: 1.5 T (*n* = 104; 75 patients, 29 healthy controls); 3 T (*n* = 108; 86 patients, 22 healthy controls). Native T1_spleen_ was assessed using heart-rate-independent ShMOLLI prototype sequence at rest and during adenosine stress (140 μg/kg/min, 4 min, IV) in 3 short-axis slices (basal, mid-ventricular, apical). This was compared with changes in peak splenic perfusion signal intensity (ΔSI_spleen_) and the “splenic switch-off” sign on conventional stress/rest gadolinium perfusion imaging. T1_spleen_ values were obtained blinded to perfusion ΔSI_spleen_, both were derived using regions of interest carefully placed to avoid artefacts and partial-volume effects.

**Results:**

Normal resting splenic T1 values were 1102 ± 66 ms (1.5 T) and 1352 ± 114 ms (3 T), slightly higher than in patients (1083 ± 59 ms, *p* = 0.04; 1295 ± 105 ms, *p* = 0.01, respectively). T1_spleen_ decreased significantly during adenosine stress (mean ΔT1_spleen_ ~ −40 ms), independent of field strength, age, gender, and cardiovascular diseases. While ΔT1_spleen_ correlated strongly with ΔSI_spleen_ (rho = 0.70, *p* < 0.0001); neither indices showed significant correlations with conventional hemodynamic markers (rate pressure product) during stress. By ROC analysis, a ΔT1_spleen_ threshold of ≥ −30 ms during stress predicted the “splenic switch-off” sign (AUC 0.90, *p* < 0.0001) with sensitivity (90%), specificity (88%), accuracy (90%), PPV (98%), NPV (42%).

**Conclusions:**

Adenosine stress and rest splenic T1-mapping is a novel method for assessing stress responses, independent of conventional hemodynamic parameters. It enables prediction of the visual “splenic switch-off” sign without the need for gadolinium, and correlates well to changes in splenic signal intensity during stress/rest perfusion imaging. ΔT1_spleen_ holds promise to facilitate optimization of stress responses before gadolinium first-pass perfusion CMR.

**Electronic supplementary material:**

The online version of this article (doi:10.1186/s12968-016-0318-2) contains supplementary material, which is available to authorized users.

## Background

Adenosine stress perfusion cardiovascular magnetic resonance (CMR) accurately detects myocardial ischemia and guides clinical decision-making [1, 2]. However, perfusion CMR has a reported false-negative rate of between 5 and 16% [2–4], which may lead to suboptimal management strategies. In the absence of poor image quality, inadequate adenosine stress response is the commonest cause of false-negative perfusion scans [4], because conventional hemodynamic markers of stress response, such as heart rate and systolic blood pressure, are unreliable predictors of myocardial vasodilatation and the achievement of maximal hyperemia [5].

Recently, the “splenic switch-off” sign was proposed as a CMR marker of adequate adenosine stress. It describes visually reduced splenic perfusion during stress imaging (spleen appears dark) compared to rest imaging (spleen appears bright) [6], and in retrospective analyses, failed splenic switch-off was more commonly observed in false-negative perfusion scans than true-negatives [6]. The physiological basis for this phenomenon is that splenic blood volume reduces significantly during exercise, due to splanchnic blood redistribution [7, 8], and can manifest as splenic “disappearance” on nuclear imaging [9]. The degree of splenic blood volume reduction is proportional to exercise workload [7], independent of cardiac output [7], and is related to adenosine-mediated splenic vasoconstriction [10, 11]. More recently, splenic switch-off has been shown to relate to higher myocardial T2 values during dipyridamole stress, further suggesting a connection between splenic and myocardial vascular biology [12].

A key limitation of splenic switch-off is that it can only be assessed after gadolinium first-pass perfusion imaging [6], at which point it is too late to optimize stress adequacy [13]. Repetition of inadequately stressed images would require a wait-period (10–15 min) for gadolinium “wash-out” from the LV cavity to optimize myocardial-blood contrast during the subsequent (no longer first-pass) stress perfusion imaging, leading to longer scan durations, and exposes patients to additional adenosine and contrast agents [6]. Therefore, a method which can determine stress adequacy and offer opportunities for pre-emptive stress response optimization *before* gadolinium first-pass perfusion imaging is highly desirable.

Native T1-mapping enables quantitative characterization of tissue blood volumes without the need for gadolinium-based contrast agents (GBCA) [14–16], and offers the potential to assess stress responses before GBCA first-pass perfusion. T1 (proton spin-lattice) relaxation time is a magnetic property of tissues measured in milliseconds [14], and each tissue type, including the spleen, has its own normal range of T1 values [14]. T1 is sensitive to changes in tissue water content or blood volume [15–19], and we recently showed that normal myocardial T1 increases by 6% during adenosine vasodilatory stress, due to expansions in myocardial blood volume [15, 16]. Furthermore, stress-T1 appears sensitive to changes in normal, ischemic and infarcted myocardium, without the need for GBCA [15]. Contrary to its vasodilatory effects in the myocardium, adenosine causes splenic *vasoconstriction*, reducing the splenic blood volume, and thus expected to lower the splenic T1 (T1_spleen_). Conveniently, the spleen is typically visible on stress perfusion CMR and can be inspected without additional planning.

This study sought to evaluate stress and rest T1_spleen_ as a gadolinium-free CMR marker of adenosine stress responses by comparing with the existing “splenic switch-off” sign and hemodynamic markers. We hypothesized that: (i) T1_spleen_ will decrease significantly from resting values during adenosine stress, due to splenic blood volume reductions and; (ii) stress-related changes in T1_spleen_ (ΔT1_spleen_) correlate to changes in splenic perfusion on CMR (the “splenic switch-off” sign), but without the need for GBCA.

## Methods

All study procedures received favourable opinions from local ethics committees, and all subjects gave written informed consent.

### Study population

To establish the relationship between T1_spleen_ and splenic perfusion/switch-off, retrospective analysis was performed on CMR scans of 212 subjects; 104 subjects had CMR at 1.5 T (Magnetom Avanto, Siemens Healthcare, Erlangen, Germany) and 108 subjects had CMR at 3 T (Magnetom Trio a Tim system, Siemens Healthcare, Erlangen, Germany). The 1.5 T population had 75 patients (*n* = 36 known coronary artery disease [CAD], *n* = 39 Atrial Fibrillation [AF]) and 29 healthy controls; the 3 T population had 86 patients (*n* = 22 known CAD, *n* = 23 Type II Diabetes Mellitus [DM], *n* = 21 Severe Aortic Stenosis [AS], *n* = 20 Hypertrophic Cardiomyopathy [HCM]) and 22 healthy controls. Healthy controls had no history of cardiovascular disease, were not on regular medications, and had normal electrocardiograms.

### CMR protocol

All subjects avoided adenosine antagonizers (e.g. caffeine) for ≥24 h before CMR. T1-mapping was performed using the Shortened Modified Look-Locker Inversion recovery (ShMOLLI) prototype sequence (WIP 561 and 448C) with inline map generation, which uses 9-heartbeats breath-holds per T1-map acquisition and enables on-screen image reconstruction within 10 s [14].

Native T1-maps were acquired at rest and during peak adenosine stress (140 μg/kg/min, 4 min, IV) in short-axis (basal, mid-ventricular, apical) slices, followed immediately by first-pass perfusion imaging on matching slices during peak stress, with an IV bolus of GBCA (0.03 mmol/kg at 6 ml/s; Dotarem, Guerbet, Villepinte, France) and saline flush (15 ml at 6 ml/s) [15, 16]. Matching rest perfusion images were acquired >15 min after stress perfusion and adenosine discontinuation to allow sufficient time for contrast washout [15, 16].

### T1-mapping analysis

**Fig. 1:**
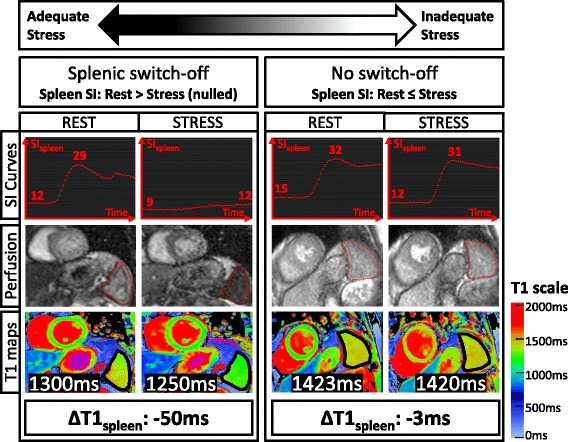
Representative stress and rest splenic first-pass gadolinium perfusion and native T1-maps. Signal intensity (*SI*) curves represent splenic perfusion SI (*y-axis, arbitrary units*) over time (*x-axis, 50–60 s*). The maximum and minimum SI_spleen_ are as indicated. Splenic regions of interests on perfusion images and T1-maps are outlined in red and black, respectively. Mean native T1_spleen_ and stress changes (*ΔT1*_*spleen*_) are as labelled. 3 T images were used for illustration (*observed ΔT1*_*spleen*_ and *ΔSI*_*spleen*_ *are field strength independent*)

Separate data files containing all T1-maps were created and anonymized before analysis by an observer (>3 years of T1-mapping analysis experience) blinded to perfusion images and clinical information. T1-maps were excluded from analysis if the spleen was not clearly visible (2%), had respiratory-motion artefacts on raw Inversion-Recovery-weighted images (3%) or had suboptimal goodness-of-fit R^2^-maps (2%) [17, 20]. Overall, 738 T1-maps were included in final analysis, using dedicated in-house software MC-ROI (programmed by S.K.P. in IDL, version 6.1, Exelis Visual Information Solutions, Boulder, Colorado) [14–18, 20]. To estimate mean native T1_spleen_, regions of interest (ROIs) were manually placed on T1-maps to include as much splenic tissue as possible, avoiding partial volume effects from large splenic blood vessels and borders with neighbouring tissues (Fig. 1). ROIs were quality checked against corresponding Inversion-Recovery-weighted images and R^2^-maps. To derive thresholds suitable for direct application on the CMR console, splenic T1-reactivity to adenosine stress (ΔT1_spleen_) was expressed in absolute terms: ΔT1_spleen_ (ms) = StressT1_spleen_ – RestT1_spleen_.

### T1_spleen_ intra-scan variability assessments

Inter-slice variability in resting T1_spleen_, stress T1_spleen_ and ΔT1_spleen_ were assessed in cases where matching stress and rest T1-maps were performed in ≥2 different short-axis slice positions. To assess for intra-slice T1_spleen_ variability, we re-analyzed healthy-volunteers data from the original ShMOLLI methods paper, where T1-maps were repeated >15 min apart in the same short-axis slice within the same scan [14]. Intra-scan variability was calculated as the standard deviation of differences-from-the-mean in each individual.

### Splenic perfusion analysis

Splenic first-pass perfusion was analysed by an observer (>4 years of perfusion imaging analysis experience) blinded to T1-maps and clinical information, using CMR^42^ software (Circle Cardiovascular Imaging Inc., Calgary, Canada). Splenic ROIs were placed on stress and rest perfusion images with frame-by-frame manual correction for artefacts and respiratory motion, to generate curves showing mean splenic signal intensity (SI, arbitrary units) changes over time (50–60s). Peak splenic perfusion SI (SI_spleen_) was estimated as the numerical difference between baseline-SI and maximal-SI during splenic first-pass perfusion as previously described [6]. Adenosine-induced changes in SI_spleen_ compared to rest were expressed in percentages: ΔSI_spleen_ (%) = (StressSI_spleen_ – RestSI_spleen_) ÷ RestSI_spleen_ × 100%.

Splenic switch-off on perfusion imaging was visually assessed by 2 independent observers (>3 years clinical CMR perfusion experience). In the 5/212 cases where the 2 observers disagreed, adjudication was sought from a 3^rd^ independent observer (Fig. 1). Perfusion images were graded as previously described [6]: either displaying *splenic switch-off* (the spleen on rest imaging is clearly brighter than on stress imaging), or *no switch-off (*the spleen on rest imaging is of similar brightness compared to stress imaging).

### Statistical analysis

Data are reported as mean ± SD, tests are 2-tailed and parametric, based on Kolmogorov-Smirnov normality-checks. Differences in individual characteristics were tested using Student’s *t*-tests, paired within individuals (e.g. stress vs rest T1_spleen_) and unpaired between groups (e.g. ∆T1_spleen_ in controls vs patients). Comparisons between ≥3 data groups were assessed using analysis of variance (ANOVA) with Bonferroni-corrected post-hoc method. Linear correlations were assessed using Pearson’s correlation coefficient (R) and non-linear correlations were assessed using Spearman’s rank correlation coefficient (rho). Intra-scan variability and inter-observer reproducibility of rest/stress T1_spleen_ and ΔT1_spleen_ were assessed using the Intra-class correlation coefficient (ICC), reporting 95% confidence intervals. The performance of ΔT1_spleen_ for replicating splenic switch-off was assessed using receiver-operating characteristics (ROC) curves [21], reporting area-under-the-curve (AUC ± SEM), and also sensitivity, specificity, diagnostic accuracy, positive predictive values (PPV) and negative predictive values (NPV), with 95% confidence intervals (CI). All analyses were performed on single measures per-subject, using MedCalc 12.7.8 (MedCalc Software, Ostend, Belgium). *P* < 0.05 denotes statistical significance.

## Results

### Subject characteristics

Subject characteristics are summarised in Table 1. All subjects experienced at least one adenosine-related symptoms (e.g. chest-tightness, dyspnoea, flushing) [13], and >10 bpm increase in heart rate (HR) during adenosine stress, compared to rest. Significant blood pressure response (>10 mmHg SBP decrease during stress) was observed in 50% of subjects.

**Table 1:** Characteristics of study subjects: healthy controls and patients (*n* = 212)

	1.5 T Controls *n* = 29	1.5 T Patients *n* = 75	3 T Controls *n* = 22	3 T Patients *n* = 86
Age (years)	54 ± 17	65 ± 9*	43 ± 12	60 ± 14*
Men (%)	21 (72)	58 (77)	13 (59)	58 (67)
Body mass index (kg/m^2^)	25 ± 4	28 ± 5	26 ± 3	28 ± 4
Hematocrit	0.42 ± 0.03	0.43 ± 0.03	0.42 ± 0.04	0.42 ± 0.03
CMR hemodynamic data				
Rest HR (bpm)	66 ± 11	62 ± 15	62 ± 12	65 ± 10
Stress HR (bpm)	96 ± 15	79 ± 15*	95 ± 12	91 ± 14
Rest SBP (mmHg)	133 ± 21	139 ± 19	127 ± 14	136 ± 19
Stress SBP (mmHg)	127 ± 19	133 ± 19	122 ± 16	126 ± 19
Rest RPP (bpm.mmHg)	8800 ± 2200	8600 ± 2200	7600 ± 1700	8700 ± 2000
Stress RPP (bpm.mmHg)	12,200 ± 2600	10,500 ± 2500*	12,000 ± 2200	11,700 ± 2700
Adenosine symptoms	29 (100)	75 (100)	22 (100)	86 (100)
Co-morbidities				
Current smoker	3 (10)	2 (3)	2 (9)	12 (14)
Ex-smoker	3 (10)	21 (28)	3 (14)	21 (24)
Hypertension	-	28 (37)	-	24 (28)
Hyperlipidemia	-	23 (31)	-	23 (27)
Stroke/TIA	-	2 (3)	-	2 (2)
Medications				
Aspirin	-	36 (48)	-	35 (41)
Beta-blocker	-	34 (45)^#^	-	19 (22)
ACEi/ARB	-	36 (48)	-	39 (45)
Statin	-	31 (41)^#^	-	44 (51)
Nitrates	-	3 (4)		4 (5)
CCB (non-DHP)	-	13 (17)^#^	-	0 (0)
CCB (DHP)		4 (5)		7 (8)

Values are *n* (%) or mean ± SD

*Abbreviations*: *RPP* rate pressure product, *TIA* transient ischemic attack, *ACEi* angiotensin-converting enzyme inhibitors, *ARB* angiotensin receptor blockers, *CCB* calcium channel antagonist, *DHP* dihydropyridine

*p < 0.05 compared to controls of corresponding field strength (1.5 T or 3 T). ^#^ *p* < 0.05 for comparisons between patient groups (1.5 T vs 3 T)

Mean stress HR was lower in 1.5 T patients compared to other subjects, despite similar resting HR, likely due to more frequent beta-blocker and non-dihydropyridine calcium channel antagonist administration in these patients (all AF/CAD, Table 1).

### Stress and rest T1_spleen_ in controls and patients

In healthy controls, mean resting T1_spleen_ values were 1102 ± 66 ms (1.5 T) and 1352 ± 114 ms (3 T), which decreased significantly during adenosine stress at 1.5 T (ΔT1_spleen_: −40 ± 25 ms, *p* < 0.0001) and 3 T (ΔT1_spleen_: −43 ± 31 ms, *p* < 0.0001). Patients had slightly lower resting T1_spleen_ compared to controls at 1.5 T (1083 ± 59 ms vs. 1102 ± 66 ms, *p* = 0.04), and this pattern was more pronounced at 3 T (1295 ± 105 ms vs. 1352 ± 114 ms, p = 0.01). Despite these observed resting T1_spleen_ differences, ΔT1_spleen_ was comparable between patients and controls, at 1.5 T (−44 ± 21 ms vs. −40 ± 25 ms, *p* = 0.43) and 3 T (−44 ± 26 ms vs. −43 ± 31 ms, *p* = 0.93; Table 2). In controls, there was a strong correlation between stress ΔT1_spleen_ (mean −4.1 ± 1.5%) and ΔT1_myocardium_ (mean 5.9 ± 1.8%), r = −0.72, *p* < 0.001. See Additional file 1 for more details.

**Table 2:** Rest and stress splenic T1 in healthy controls and patients

	1.5 T Controls	1.5 T Patients	*p*-value	3 T Controls	3 T Patients	*p*-value
Rest T1_spleen_ (ms)	1102 ± 66	1083 ± 59	0.04	1352 ± 114	1295 ± 105	0.01
Stress T1_spleen_ (ms)	1061 ± 68	1039 ± 55	0.02	1308 ± 114	1253 ± 112	0.01
ΔT1_spleen_ (ms)	−40 ± 25	−44 ± 21	0.43	−43 ± 31	−44 ± 26	0.93

ΔT1_spleen_ = StressT1_spleen_ – RestT1_spleen_

In pooled analysis, ΔT1_spleen_ did not appear to be significantly affected by field strength (1.5 T vs. 3 T: −43 ± 22 ms vs. −42 ± 27 ms, p = 0.89), gender (male vs. female: −40 ± 23 ms vs. −47 ± 28 ms, *p* = 0.09), age (R = 0.10, *p* = 0.14, range 21–89 years) or the type of cardiovascular diseases (1.5 T CAD −42 ± 20 ms vs. 3 T CAD −40 ± 25 ms vs. AF −46 ± 22 ms vs. HCM −43 ± 28 ms vs. AS −43 ± 21 ms vs. DM −43 ± 32 ms, p = 0.54). In addition, ΔT1_spleen_ was not significantly affected by medication in patients (supplementary material in Additional file 2).

### T1_spleen_ intra-scan variability

Inter-slice intra-scan variability (assessable in 96 subjects) was within ±19 ms for resting T1_spleen_, ±18 ms for stress T1_spleen_, and ±10 ms for ΔT1_spleen_. Re-analysis of the original ShMOLLI cohort (spleen visible in 9/10 cases), revealed an intra-slice intra-scan repeat variability of T1_spleen_ of ±9 ms, ICC: 0.98 (95% confidence interval 0.93 to 0.99) [14]. ΔT1_spleen_ was derived by a second independent blinded observer in 45 subjects (20 controls; 25 patients: 5 CAD, 5 AF, 5 DM, 5 AS, 5 HCM), which yielded an ICC of 0.87 (95% confidence interval: 0.76 to 0.93). The Bland-Altman plot for inter-observer variability is shown in supplementary material (Additional file 3).

### Associations between splenic perfusion, T1 and rate pressure product (RPP)

**Fig. 2:**
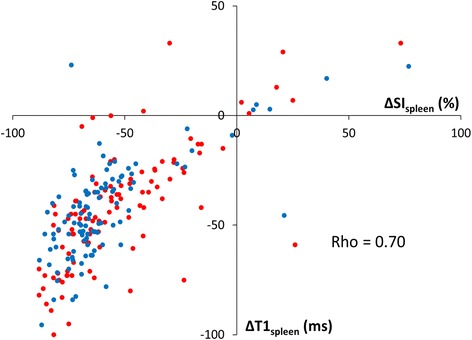
Correlation between stress-induced reductions in peak splenic signal intensity (*ΔSI*_*spleen*_) and splenic native T1 (*ΔT1*_*spleen*_). Pooled data of controls and patients at 1.5 T (*blue*) and 3 T (*red*), represented on per-subject basis (*n* = 212). Spearman’s rank correlation coefficient (*Rho*) = 0.70, *p* < 0.0001

By semi-quantitative analysis, peak splenic perfusion SI (SI_spleen_) decreased significantly with adenosine stress compared to rest, with no differences between controls and patients, or across field strengths (Table 3). ΔSI_spleen_ correlated strongly with ΔT1_spleen_ (rho = 0.70, *p* < 0.0001, Fig. 2). In contrast, ΔSI_spleen_ and ΔT1_spleen_ did not demonstrate significant correlations with stress-induced changes in RPP (*R* = 0.04, *p* = 0.60; *R* = 0.06, *p* = 0.38, respectively).

**Table 3:** Peak splenic perfusion signal intensity (SI_spleen_) at rest and during adenosine stress (ΔSI_spleen_) in healthy controls and patients

	1.5 T Controls	1.5 T Patients	3 T Controls	3 T Patients	*p*-value
Rest SI_spleen_ (au)	26 ± 8	24 ± 11	29 ± 13	27 ± 13	0.16
Stress SI_spleen_ (au)	11 ± 5	11 ± 5	13 ± 12	12 ± 8	0.10
ΔSI_spleen_ (%)	−58 ± 23	−54 ± 22	−56 ± 28	−52 ± 30	0.51

*Abbreviations*: *Au* arbitrary units

*P*-values derived using ANOVA with Bonferroni post-hoc method

### Visual splenic switch-off assessment – relationships with perfusion, quantitative T1_spleen_ and hemodynamic parameters

**Fig. 3:**
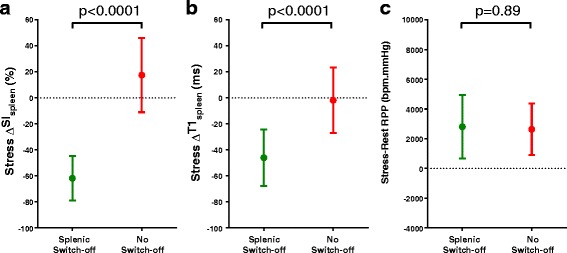
Relations between different markers of stress adequacy. Subjects with the “splenic switch-off” sign had greater stress-induced reductions in **a** *gadolinium-based* splenic perfusion (ΔSI_spleen_, same technique) and **b** *gadolinium-free* splenic T1 (ΔT1_spleen_, different technique) compared to subjects with no switch-off. There was no difference in stress-induced **c** *hemodynamic* changes in rate pressure product (*RPP*) between the splenic switch-off and the no switch-off subjects. Data are mean ± 1SD

Subjects with visual splenic switch-off had greater stress ΔSI_spleen_ and ΔT1_spleen_ values compared to those with no switch-off (Table 4 and Fig. 3). In contrast, there were no significant differences in stress-related haemodynamic changes (HR, SBP, RPP) between subjects with splenic switch-off and no switch-off (Table 4 and Fig. 3).

**Table 4:** Stress-induced changes in peak splenic perfusion signal intensity (ΔSI_spleen_), T1 (ΔT1_spleen_) and hemodynamic parameters for visually assessed “splenic switch-off” sign

	Splenic Switch-off	No Switch-off	*p*-value
All subjects *n* = 212	196 (92)	16 (8)	-
Healthy volunteers *n* = 51	49 (96)	2 (4)	-
Patients *n* = 161	147 (91)	14 (9)	-
ΔSI_spleen_ (%)	−62 ± 17	17 ± 29	<0.0001
ΔT1_spleen_ (ms)	−46 ± 22	−2 ± 25	<0.0001
Stress hemodynamic changes			
Δ heart rate (bpm)	19 ± 9	20 ± 12	0.83
Δ SBP (mm. Hg)	−8 ± 22	−10 ± 19	0.76
Δ RPP (bpm.mmHg)	2800 ± 2100	2600 ± 1700	0.89

Values are *n* (%) or mean ± SD

*Abbreviations*: *Bpm* beats per minute, *SBP* systolic blood pressure, *RPP* rate pressure product

### ROC analysis of native ΔT1_spleen_ for replicating gadolinium-based “splenic switch-off”

**Fig. 4:**
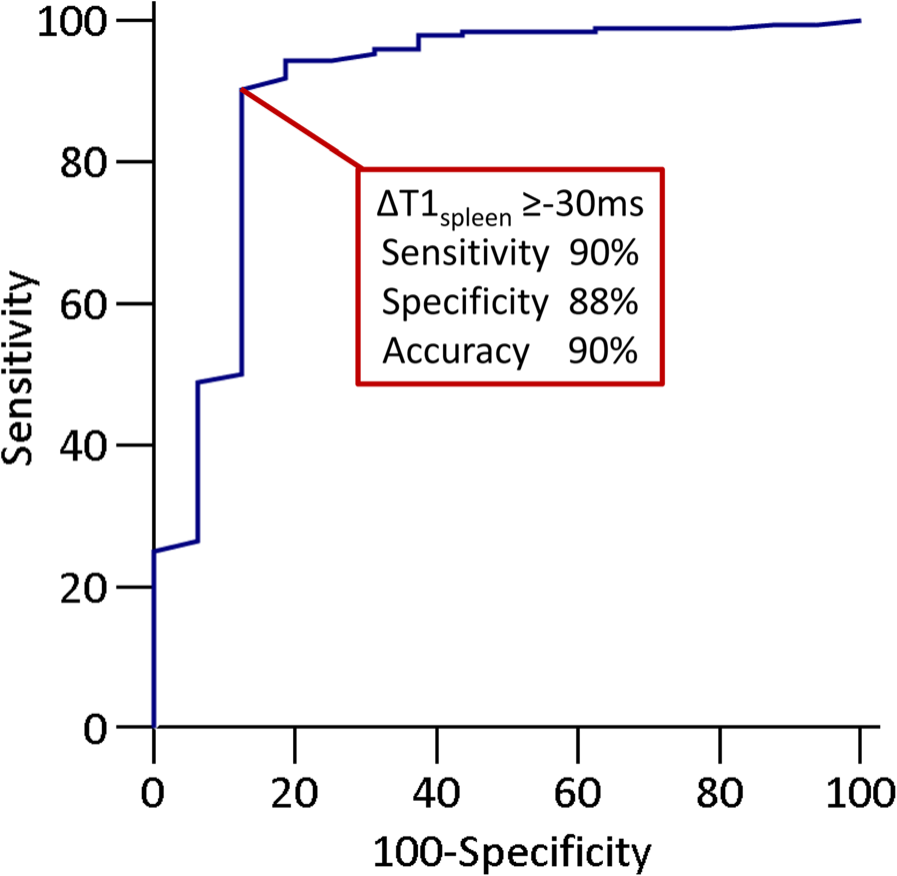
ROC curves of native ΔT1_spleen_ for replicating the gadolinium-based “splenic switch-off” sign. A ΔT1_spleen_ threshold of ≥ −30 ms replicated the “splenic switch-off” sign (AUC 0.90 ± 0.05, *p* < 0.0001), with high sensitivity 90%, specificity 88% and diagnostic accuracy 90%

ROC analysis using visual splenic switch-off as reference standard (true positives = splenic switch-off, true negatives = no switch-off) yielded an AUC of 0.90 ± 0.05 (*p* < 0.0001, Fig. 4). A ΔT1_spleen_ threshold of ≥ −30 ms during adenosine stress replicated visual splenic switch-off with a sensitivity of 90% (95% CI: 85–94%, *p* < 0.0001), specificity 88% (95% CI: 62–98%, *p* < 0.0001), diagnostic accuracy 90% (95% CI: 84–96%, *p* < 0.0001), PPV 98% (95% CI: 96–100%, *p* < 0.0001) and NPV 42% (95% CI: 26–61%, *p* < 0.0001).

## Discussion

**Fig. 5:**
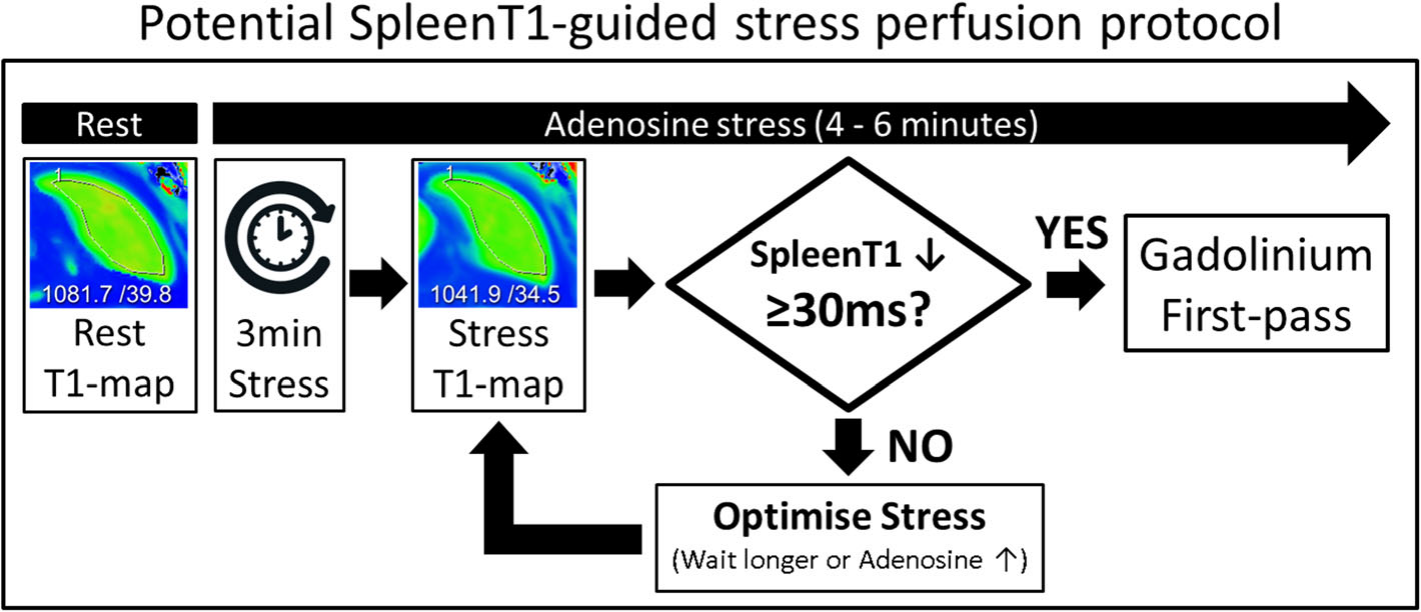
Potential splenic ΔT1_spleen_-guided protocol for real-time assessment and optimization of stress adequacy before gadolinium perfusion. Practical T1_spleen_ assessment using ShMOLLI typically takes around 30 s: breath-hold instructions (*5 s*), T1-map acquisition over 9-heart-beats (~10 s, shorter with higher stress heart rates), on-screen image reconstruction (*5*–*10 s*), splenic-ROI placement directly on CMR console screen by the operator (*5 s*) followed by immediate display of T1_spleen_/SD estimations (as indicated). The ability of this protocol to improve the quality of stress responses deserves validation in future studies

This proof of principle study demonstrated that T1_spleen_ decreases significantly during adenosine stress compared to baseline. The magnitude of the stress-induced T1_spleen_ response (ΔT1_spleen_) is strongly correlated with splenic perfusion attenuation (ΔSI_spleen_). From a clinical viewpoint, a native ΔT1_spleen_ threshold of ≥ −30 ms accurately replicated the “splenic switch-off” sign with a high positive predictive value of 98% and offers the potential to assess adenosine stress adequacy before GBCA first-pass perfusion imaging. From a practical viewpoint, assessment of T1_spleen_ takes ~30 s (Fig. 5), which means it can be repeated as necessary “on-the-fly”, to guide adenosine dosage up-titrations and optimize stress responses before injection of contrast agents (example protocol in Fig. 5). This pre-gadolinium approach may be advantageous over the retrospective and potentially gadolinium dose-sensitive splenic switch-off method for improving the quality of stress responses before first-pass perfusion imaging, which deserves further investigation in future studies to determine whether it decreases the number of false negative perfusion scans [6].

### Stress/rest T1_spleen_ as a marker of adenosine stress response

Patients had lower resting native T1_spleen_ values compared to controls. This may be related to the presence of co-morbidities in patients, such as hypertension and peripheral vascular disease, which may induce peripheral vasoconstriction, with expected reductions in resting organ blood volumes and T1_spleen_ values. This observation deserves further investigation in larger future studies. Native T1-relaxation times of tissues are prolonged by increased blood volume (i.e. water content) [14, 15, 22]. Adenosine causes splenic artery vasoconstriction and reduced blood volume [6–11], which shortens splenic T1-relaxation times. This is supported by our observation of significantly lower T1_spleen_ during adenosine stress compared to rest, in both controls and patients. The stress ΔT1_spleen_ was not significantly affected by different field strengths, age, gender and cardiovascular diseases, likely reflecting reproducible T1-estimations in this study [14, 15, 22].

The correlation between stress ΔT1_spleen_ and ΔT1_myocardium_ in normal controls suggests the vasoconstrictor effect of adenosine on the spleen is associated with vasodilatory effects in the myocardium. For the relationship between myocardial and splenic stress T1 in patients with cardiovascular disease, larger ongoing studies will offer reference ranges for ΔT1 in disease, and resolve the separate effects of regional myocardial differences and medication on stress T1 reactivity.

The observed strong correlation between ΔT1_spleen_ and ΔSI_spleen_ suggests that stress-induced changes in splenic blood volume are related to blood flow, which is regulated by alterations in the adenosine-mediated splenic arterial tone [10, 11]. The lack of significant correlation between ΔSI_spleen_ or ΔT1_spleen_ with rate pressure product is consistent with existing evidence showing dissociation between imaging and hemodynamic markers of stress response [5, 6], and further suggests that stress responses during clinical CMR cannot be reliably assessed using hemodynamic observations alone [5]. This deserves further investigation.

A threshold of ≥30 ms decrease in T1_spleen_ replicated complete splenic switch-off with a high positive predictive value of 98%. The intra-scan variability in T1_spleen_ (inter-slice: ±10 ms; intra-slice: ±9 ms) was 3-times less than this proposed threshold ≥30 ms drop, with excellent T1-fit as evident on quality control R^2^-maps, despite the lack of dedicated image optimization (e.g. shimming) over the spleen. For stress T1_spleen_ responses <30 ms, further work is needed to determine whether adenosine dose-increments or waiting longer with the same infusion rate may improve the confidence of stress responses, and impact on diagnostic performance of stress CMR for the diagnosis of ischemia.

### Limitations and future directions

This proof-of-concept study is based on ShMOLLI T1_spleen_ values derived from short-axis slices planned for myocardial perfusion CMR imaging; the spleen was not visible in a small proportion of T1-maps (~2%), and future applications of splenic T1-mapping may benefit from a dedicated image planned through the spleen. Rapid on-scanner T1-map reconstructions, with the immediate availability of goodness-of-fit measures (such as R^2^-maps), are imperative to enable practical “on-the-fly” repetition of reliable T1_spleen_ estimations to guide stress response optimization (Fig. 5). Given the overall excellent R^2^-maps over the spleen and the narrow T1_spleen_ ranges obtained, data in this study suggest that stress/rest splenic T1-mapping can be feasibly included in CMR protocols without major technical adjustments. Practical in-vivo T1-estimations are method-dependent, and demonstrate increasingly discrepant heart rate dependencies at longer T1-values [23]. Therefore, results achieved with ShMOLLI, in particular the splenic T1-thresholds replicating splenic switch-off, should be interpreted with care before directly translating to other T1-mapping techniques. Choosing methods that can withstand dynamic HR-variations and tachycardia without significant HR-dependencies is therefore paramount when performing stress-T1 studies. The gadolinium-based splenic switch-off sign is only seen with non-selective adenosine receptor agonists (dipyridamole and adenosine), but was absent with cardio-selective vasodilators (e.g. regadenoson) or inotropic agents (e.g. dobutamine) [6]. Further work is needed to elucidate stress T1_spleen_ responses using pharmacological agents other than adenosine and during physical exercise. Patients in this study were unselected for diseases known to affect splenic blood volumes, e.g. venous portal hypertension, hematological malignancies and systemic inflammation; thus, further studies to characterize the effects of these diseases on T1_spleen_ will help to determine the general applicability of this technique. While we identified a cut-off of ≥30 ms drop in T1_spleen_ during stress for replicating complete splenic switch-off, the clinical utility of this threshold for detecting *true* stress adequacy needs to be validated against false-negative perfusion scans, determined by comparison to invasive coronary angiography and pressure-wire based assessments of functional ischemia, such as fractional flow reserve. This is topic of ongoing work.

## Conclusions

Adenosine stress and rest splenic T1-mapping is a novel method for assessing stress responses, independent of conventional hemodynamic parameters. It enables prediction of the visual “splenic switch-off” sign without the need for gadolinium, and correlates well to changes in splenic signal intensity during stress/rest perfusion imaging. ΔT1_spleen_ holds promise to facilitate optimization of stress responses before gadolinium first-pass perfusion CMR.

## Additional files

Additional file 1: Figure S1. Description of data: Correlation between adenosine stress ΔT1_spleen_ and ΔT1_myocardium_ in 51 healthy controls. Data are presented per-subject. (DOCX 26 kb)
Additional file 2: Table S1. Description of data: Effect of medication on ΔT1_spleen_ in patients with cardiovascular disease. ACE: angiotensin converting enzyme; ARB: angiotensin reception blocker; CCB: calcium channel blockers; DHP: dihydropyridine. (DOCX 13 kb)
Additional file 3: Figure S2. Description of data: Bland Altman plot of ΔT1_spleen_ estimation by 2 independent blinded observers. (DOCX 38 kb)

## Abbreviations

CAD: Coronary artery disease
CMR: Cardiovascular magnetic resonance
ECG: Electrocardiogram
GBCA: Gadolinium-based contrast agents
ROC: Receiver-operating characteristics
ShMOLLI: Shortened modified look-locker inversion recovery
SI_spleen_: Peak splenic signal intensity
T1_spleen_: Mean native splenic T1 values
