# Editorial Expression of Concern: Splenic T1-mapping: a novel quantitative method for assessing adenosine stress adequacy for cardiovascular magnetic resonance

**DOI:** 10.1186/s12968-023-00935-y

**Published:** 2023-05-25

**Authors:** Alexander Liu, Rohan S. Wijesurendra, Rina Ariga, Masliza Mahmod, Eylem Levelt, Andreas Greiser, Mario Petrou, George Krasopoulos, John C. Forfar, Rajesh K. Kharbanda, Keith M. Channon, Stefan Neubauer, Stefan K. Piechnik, Vanessa M. Ferreira

**Affiliations:** 1 Oxford Centre for Clinical Magnetic Resonance Research (OCMR), Division of Cardiovascular Medicine, Radcliffe Department of Medicine, University of Oxford, Oxford, UK; 2 Siemens Healthcare GmbH, Erlangen, Germany; 3 Department of Cardiothoracic Surgery, John Radcliffe Hospital, Oxford, UK; 4 Oxford Heart Centre, John Radcliffe Hospital, Oxford, UK; 5 Division of Cardiovascular Medicine, Radcliffe Department of Medicine, University of Oxford, Oxford, UK

**Editorial Expression of Concern: Journal of Cardiovascular Magnetic Resonance (2017) 19:1** 10.1186/s12968-016-0318-2

The Editor-in-Chief would like to alert readers that concerns have been raised regarding the data in this article.

Authors Vanessa M. Ferreira, Stefan K. Piechnik and Stefan Neubauer reached out to the *Journal of Cardiovascular Magnetic Resonance* in December 2020 to report that authors Ferreira, Piechnik and Wijesurendra had re-analyzed the available data and were unable to reproduce some of the results reported in this article.

Thorough  reanalysis of available data found that the strong links between the splenic signal intensity change and delta spleen T1, and the ability of delta spleen T1 to predict the splenic switch-off sign, could not be independently reproduced, although the general observations reported in Table 2 (that the spleen T1 decreases significantly in response to Adenosine) and inter-observer reproducibility of delta spleen T1 could be reproduced.

As a result of the information available, the Editor-in-Chief advises readers to interpret the results of this publication with due caution.

Authors Rohan S. Wijesurendra, Rina Ariga, Eylem Levelt, Stefan Neubauer, Stefan K. Piechnik and Vanessa M. Ferreira agree to this Expression of Concern. Authors Alexander Liu, Masliza Mahmod, Andreas Greiser, Mario Petrou, George Krasopoulos, John C. Forfar, Rajesh K. Kharbanda and Keith M. Channon have not responded to correspondence from the editor regarding this Expression of Concern.

