# Supplementary material for: Splenic T1-mapping: a novel quantitative method for assessing adenosine stress adequacy for cardiovascular magnetic resonance
Source: J Cardiovasc Magn Reson. 2017 Jan 13;19:1. doi: 10.1186/s12968-016-0318-2 (PMC5234250; doi:10.1186/s12968-016-0318-2)
Supplement: Additional file 1: Figure S1. — Description of data: Correlation between adenosine stress ΔT1spleen and ΔT1myocardium in 51 healthy controls. Data are presented per-subject. (DOCX 26 kb) [file 12968_2016_318_MOESM1_ESM.docx]

**Additional file 1: Figure S1**

Correlation between adenosine stress ΔT1_spleen_ and ΔT1_myocardium_ in 51 healthy controls. Data are presented per-subject.
