# Supplementary material for: Splenic T1-mapping: a novel quantitative method for assessing adenosine stress adequacy for cardiovascular magnetic resonance
Source: J Cardiovasc Magn Reson. 2017 Jan 13;19:1. doi: 10.1186/s12968-016-0318-2 (PMC5234250; doi:10.1186/s12968-016-0318-2)
Supplement: Additional file 2: Table S1. — Description of data: Effect of medication on ΔT1spleen in patients with cardiovascular disease. ACE: angiotensin converting enzyme; ARB: angiotensin reception blocker; CCB: calcium channel blockers; DHP: dihydropyridine. (DOCX 13 kb) [file 12968_2016_318_MOESM2_ESM.docx]

**Additional file 2: Table S1**

|  | Mean ΔT1_spleen_ values | |  |
| --- | --- | --- | --- |
|  | YES | NO | p Value |
| Aspirin | -43±23 | -43±22 | 0.96 |
| Beta blocker | -42±21 | -45±24 | 0.61 |
| ACEi/ARB | -42±25 | -46±18 | 0.34 |
| Statin | -44±21 | -43±25 | 0.74 |
| Nitrates | -43±14 | -43±23 | 0.93 |
| CCB (non-DHP) | -40±24 | -45±22 | 0.24 |
| CCB (DHP) | -43±21 | -44±23 | 0.85 |

Effect of medication on ΔT1spleen in patients with cardiovascular disease. ACE: angiotensin converting enzyme; ARB: angiotensin reception blocker; CCB: calcium channel blockers; DHP: dihydropyridine.
