# Supplementary material for: Splenic T1-mapping: a novel quantitative method for assessing adenosine stress adequacy for cardiovascular magnetic resonance
Source: J Cardiovasc Magn Reson. 2017 Jan 13;19:1. doi: 10.1186/s12968-016-0318-2 (PMC5234250; doi:10.1186/s12968-016-0318-2)
Supplement: Additional file 3: Figure S2. — Description of data: Bland Altman plot of ΔT1spleen estimation by 2 independent blinded observers. (DOCX 38 kb) [file 12968_2016_318_MOESM3_ESM.docx]

**Additional file 3: Figure S2**


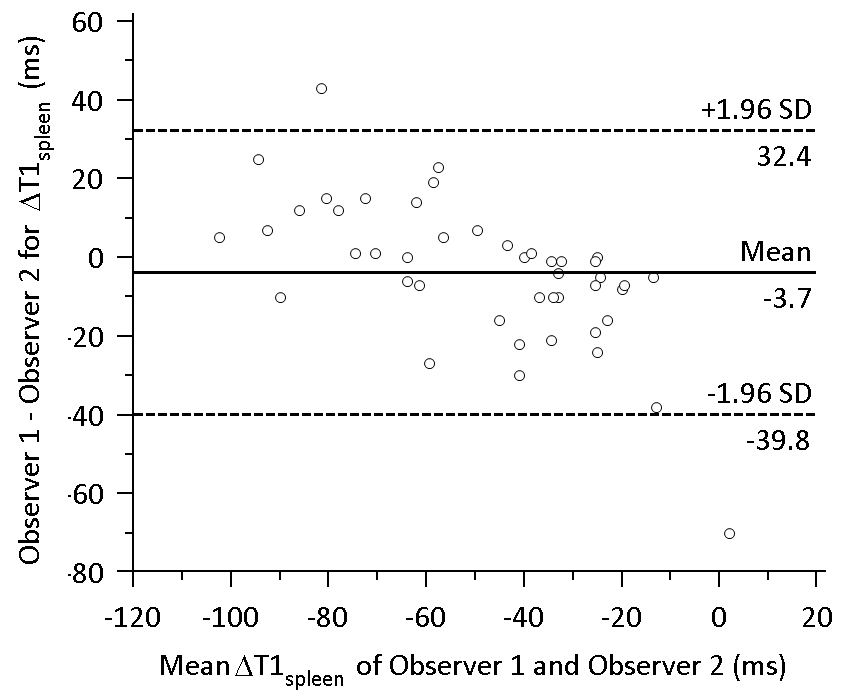


Bland Altman plot of ΔT1_spleen_ estimation by 2 independent blinded observers.
